# The ‘very moment’ when UDG recognizes a flipped-out uracil base in dsDNA

**DOI:** 10.1038/s41598-025-91705-6

**Published:** 2025-03-07

**Authors:** Vinnarasi Saravanan, Nessim Raouraoua, Guillaume Brysbaert, Stefano Giordano, Marc F. Lensink, Fabrizio Cleri, Ralf Blossey

**Affiliations:** 1 University of Lille, CNRS, UMR8576, Unité de Glycobiologie Structurale et Fonctionnelle (UGSF), F-59000 Lille, France; 2 CNRS, Centrale Lille, Univ. Polytechnique Hauts-de-France, UMR 8520 - IEMN - Institut d’Electronique, de Microélectronique et de Nanotechnologie, University of Lille, 59000 Lille, France; 3 Institut d’Electronique, de Microélectronique et de Nanotechnologie (IEMN CNRS UMR8520) and Département de Physique, University of Lille, 59652 Villeneuve d’Ascq, France

**Keywords:** Biophysics, Computational biology and bioinformatics

## Abstract

Uracil-DNA glycosylase (UDG) is the first enzyme in the base-excision repair (BER) pathway, acting on uracil bases in DNA. How UDG finds its targets has not been conclusively resolved yet. Based on available structural and other experimental evidence, two possible pathways are under discussion. In one, the action of UDG on the DNA bases is believed to follow a ‘pinch-push-pull’ model, in which UDG generates the base-flip in an active manner. A second scenario is based on the exploitation of bases flipping out thermally from the DNA. Recent molecular dynamics (MD) studies of DNA in trinucleosome arrays have shown that base-flipping can be readily induced by the action of mechanical forces on DNA alone. This alternative mechanism could possibly enhance the probability for the second scenario of UDG-uracil interaction via the formation of a recognition complex of UDG with flipped-out base. In this work, we describe DNA structures with flipped-out uracil bases generated by MD simulations which we then subject to docking simulations with the UDG enzyme. Our results for the UDG-uracil recognition complex support the view that base-flipping induced by DNA mechanics can be a relevant mechanism of uracil base recognition by the UDG glycosylase in chromatin.

## Introduction

The recognition and the subsequent cleavage of wrongly incorporated or chemically induced uracil bases in DNA by repair enzymes is the initial step in the base-excision repair (BER) process. It is performed by the enzyme Uracil-DNA glycosylase (UDG), a protein of 313 amino acids (UniProt P13051 UNG_HUMAN) that was the first glycosylase to be structurally characterized in complex with damaged DNA^1^; for a review of structural and functional properties of the UDG superfamily of glycosylases, see^2^. UDG structures were repeatedly resolved experimentally in interaction with DNA and made available trough the Protein Data Bank (PDB, https://www.rcsb.org).

UDG initiates the BER pathway for uracil excision by hydrolyzing a glycosylic bond between a uracil base and the deoxyribose sugar in order to produce a free uracil and an abasic site in the DNA strand^2^. Human UDG also removes uracil from single-stranded DNA, but it is not active against uracil in RNA^3^. Uracil excision requires the base to be flipped out of the DNA double strand so that it can be processed in the enzyme active site^1^.

DNA glycosylases must be extremely efficient to inspect all bases in the genome ($3\times 10^9$ base pairs) and detect and repair damaged bases (around 1 in $10^6$ bases per cell daily) before the genetic code is permanently altered in the next cycle of DNA replication. Given that there are on the order of $10^5$ copies of glycosylase molecules in the nucleus, each such protein excluding redundancy should be able to sample around 70,000 base pairs of DNA in every cell cycle (about 12 to 24 h)^4^. The fact that, in practice, glycosylases can achieve their job much faster points to the existence of better than purely stochastic mechanisms.

How UDG actually finds the wrongly incorporated base is a question that cannot be conclusively answered by crystallography, as it obviously is a highly dynamic process and X-ray only allows for access to final stable states of the interacting species. In order to cleave the base, UDG must first gain access to it. Several structural studies found that different DNA repair proteins can actively flip out their target base extra-helically (the so-called ‘enzymatic’ base flipping, see e.g.^5–7^); the efficiency of the excision process is sequence-dependent as demonstrated in^5^; see also the recent work by Orndorff et al.^8^. The mechanisms involved in amino-acid assisted base flipping have received ample attention in the literature also for DNA repair proteins other than UDG, see, e.g.^9–11^. Recent single-molecule experiments have provided evidence that UDG works in a ‘scanning’ mode, i.e. processing along DNA, combined with a ‘peeking’-mode, which involves the insertion of an amino acid into the DNA stack^12^. Already before experimental evidence had been provided for hopping and sliding motions of UDG on DNA^13^, a detailed ‘pinch-push-pull’ scenario had been formulated, in which the non-specific scanning step of UDG along DNA is followed by highly specific actions of UDG on DNA^3^. In this scenario, UDG first induces a kink in the DNA backbone, allowing for the formation of specific amino acid contacts with the base, which then leads to a backbone compression that ‘pinches’ the DNA. Subsequently, the intercalation loop of UDG penetrates the minor groove and flips out the uracil base. In the final ‘pull’ step, the glycosidic bond is cleaved. Importantly, apart from the non-specific diffusion or hopping along DNA, the amino-acid-DNA interaction is actively pursued by UDG. This ‘pinch-push-pull’ scenario thus starts from an initially pristine DNA, and requires UDG to very actively search for the ‘hidden’ base^3^. There is good reason to doubt that search processes are the only, or even the dominant mechanism in locating wrong bases for excision in DNA. Based on available data from in-vitro experiments, a simple Monte Carlo (MC) random search simulation could well accommodate the experimental situation; however, it remains unrealistic by several orders of magnitude for realistically estimated chromatin densities in the eukaryotic nucleus (see Supplementary Material). Such considerations therefore motivate the investigation for alternative mechanisms, by which defect-containing DNA fragments may become highly visible, and more easily identifiable by the repair proteins compared to serendipitous random search.

It is still under debate whether the spontaneous base flipping, which normally occurs at random all along the DNA because of thermodynamical fluctuations^14–17^, could be an even stronger attractor for glycosylases and other similarly functioning enzymes^18–20^. Given the much longer lifetimes measured by NMR for extrahelical flipping (in the μs to ms range) compared to intrahelical flip, and the fact that even partially flipped bases may become accessible^21–23^, spontaneous flipping must therefore be properly investigated as a possible first-step, or possibly the very rate-limiting event, in the glycosylase search for damaged DNA sites. Base flipping in pristine DNA in fact happens stochastically and an encounter with UDG could therefore also happen randomly; it is however easy to estimate that the probability of a purely random encounter (product of two independent random events), even with a substantial concentration of UDG molecules, will be generally too low to be of practical importance. DNA in the cell nucleus, however, is subject to numerous untargeted interactions, which might indeed lead to a large increase in the rate of base flipping, compared to the thermally-assisted one, thereby largely facilitating uracil recognition by UDG. In particular, mechanical constraints acting on the DNA structure have been indicated as an additional source of localized bending, twisting and kinking of the double helix, all which are favorable conditions for increasing the base-flipping rate. Uracil differs from thymine by missing the extra methyl at C5 carbon facing the major groove, which creates a distortion in the standard B-DNA structure, and leaves room for further defect evolution, such as clustering of intrastrand cross-links^24^. It has been well established that e.g. a strong curvature of DNA drastically increases its flexibility^25^. Interestingly, in a recent work^26^ we modelled by fully-atomistic molecular dynamics (MD) simulations an array of three nucleosomes, under applied mechanical forces in a range reminding the compressive regime that cells may be typically subject during their lifetime. These simulations have shown that the resulting compression of the structure can induce kink instabilities in the linker DNA which facilitate the opening of the double-stranded linker DNA and the flipping-out of the bases^26^. This mechanical mechanism leads to the formation of localized denaturation bubbles through a mechanical twist of the DNA double helix^27^. It can therefore be concluded that the ubiquitous mechanical action on DNA could therefore present a partially or fully exposed uracil base to UDG. This observation thus raises the question whether DNA mechanics can play, among other effects, a relevant role in the recognition process of uracil bases by repair enzymes. Starting from the insights gained in^26^, in this paper we characterize the interaction of UDG with exposed uracil bases and attempt to find the instant in which the recognition complex is formed between the flipped-out base and UDG. In other words, we aim at finding the ‘very moment’ (a notion inspired by^28^) when UDG can recognize a flipped-out base without having previously intervened in its formation. Here we study this problem by a combination of the controlled induction of uracil base-flipping via MD and protein-DNA docking simulations. Our finding of this ‘very moment’ of encounter entails the question of the quality of the complex formed: we compare our simulation results to the crystal structure of the UDG-DNA complex, which is assumed as representative for the action of the enzyme on uracil.

The flipping of nucleic bases has been studied by MD simulations by several authors, see, e.g.^29–32^. Studying the spontaneous base flipping is challenging, as the free energy penalty for the extrahelical state of individual DNA bases is about 10 kcal/mol^33,34^. According to previous nuclear magnetic resonance (NMR) studies^14,35^, the lifetime of the extrahelical state of a flipped DNA base is on the order of microseconds ($\upmu \text {s}$). In contrast, the intrahelical state lasts from milliseconds to hundreds of milliseconds, depending entirely on the stability of distinct base pairs in the dsDNA. This significant difference between the timescales accessible in all-atom molecular dynamics (MD) simulations and those of base flipping makes it difficult to obtain converged statistics in computer simulations. Therefore, the rare-event computational methodology is required for spontaneous base flipping mechanism. Várnai and Lavery^29^, Huang et al.^36^ and Law and Feig^37^ have used external forces to induce individual base flipping through umbrella sampling and replica exchange simulation methodologies. In this work, we have used collective variable (CV) based metadynamics^38^ simulations to study the base-flipping mechanism.

Having the base-flipped DNA structures at hand, either from the dedicated simulations described here, or from our earlier work on the compression of trinucleosomes^26^, we use protein-DNA docking methods to study the formation of the recognition complexes as a function of the flipping of the base. Contrary to protein-protein docking, where standard methods working in most of the cases are established, protein-DNA docking requires special attention to the algorithm used, in particular when DNA bears uracil. It is even more essential to assess the efficiency of these different methods for our system as it corresponds to a non-standard case: the nature of the DNA strands that are docked to UDG here is highly altered, as the treatment done to induce base flipping is causing deformations on the strand, and the flipped-out base represents a significant conformational change of the pristine dsDNA. In order to determine the best-suited protein-DNA docking method for our case, we had to test several available algorithms, and describe in detail our quantification of the quality of the encounter complex through protein-DNA docking.

## Results

### Mechanisms of uracil base flipping and associated structural deformations

We have obtained dsDNA conformations with flipped uracil bases in two ways. The first approach makes use of the structures generated during the compression MD simulations of tricnucleosome arrays performed in our earlier work^26^. How these structures were selected and prepared for our docking simulations is discussed in “Methods”.

**Fig. 1:**
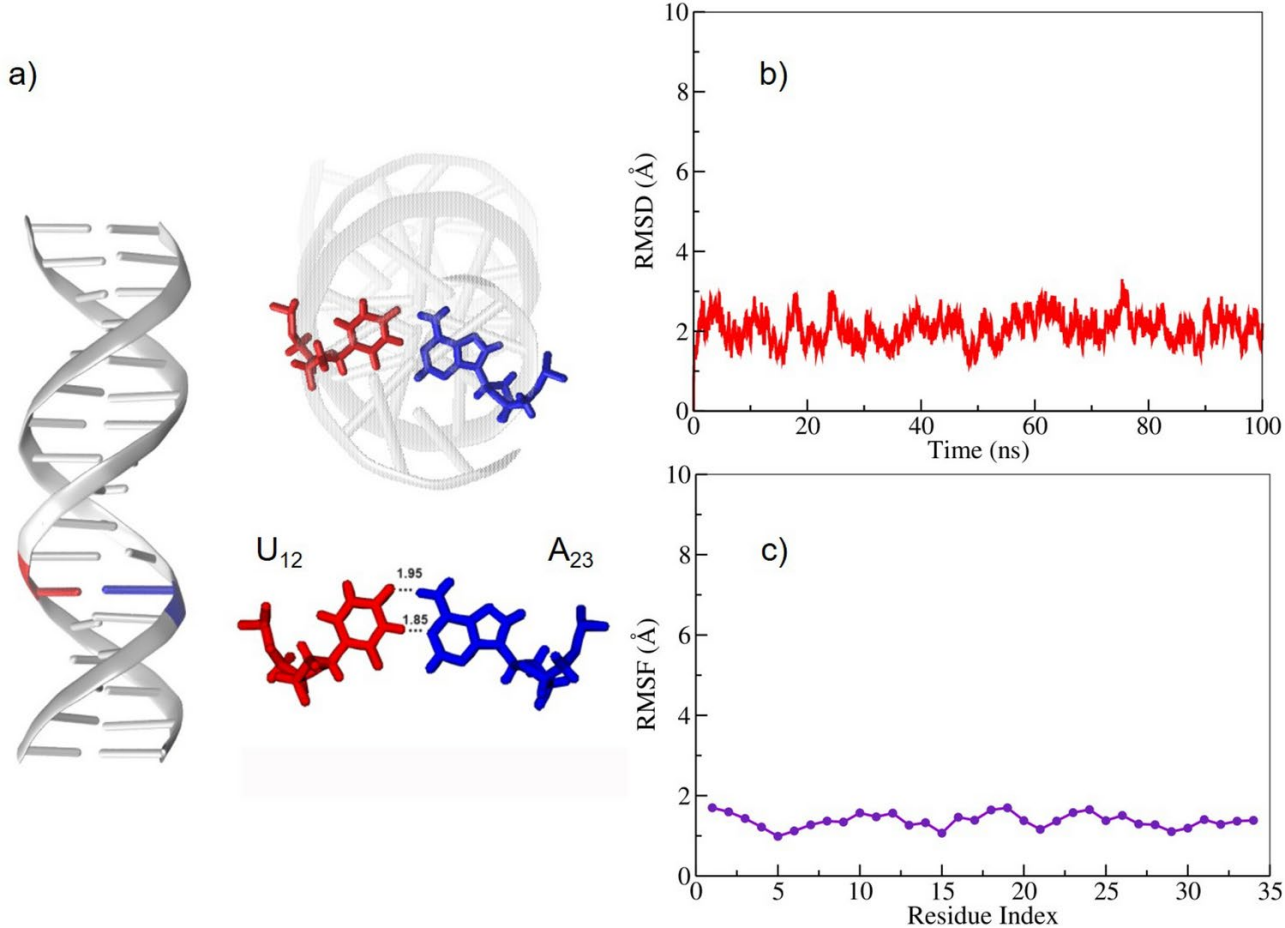
(**a**) Uracil-mutated dsDNA system with red and blue colors representing uracil and adenine residues, respectively. (**b**) Root mean square deviation (RMSD) and (**c**) Root mean square fluctutation (RMSF) of the uracil-mutated dsDNA system during the first 100 ns of the MD trajectory.

In a second approach, we have generated flipped-out bases in a controlled way from mutated dsDNA by using the metadynamics technique. For this we generated a dsDNA 17-mer in the standard B-DNA form given by the sequence $5'-\text {CAGGATGTATATATCTG}-3'$. The thymine nucleotide T at position 12 was mutated to a uracil base with its cartesian coordinates generated by the Web 3DNA server^39^. The targeted central base pair for flipping was $\text {U}_{12}$:$\text {A}_{23}$, with $\text {U}_{12}$ as the target nucleotide for base flipping; the geometry is illustrated in Fig. 1a). First, an all-atom conventional MD simulation was performed to stabilize the dsDNA system over approximately 100 ns. The root-mean-square deviation (RMSD) was used to monitor the overall stability of the dsDNA, as shown in Fig. 1b). The overall RMSD fluctuation was around 1-3 Å, which shows that the relative structural changes are very small. Moreover, the root mean-square fluctuation (RMSF) values calculated for the uracil-mutated dsDNA reveal very small fluctuations at the individual base level, see Fig. 1c).

**Fig. 2:**
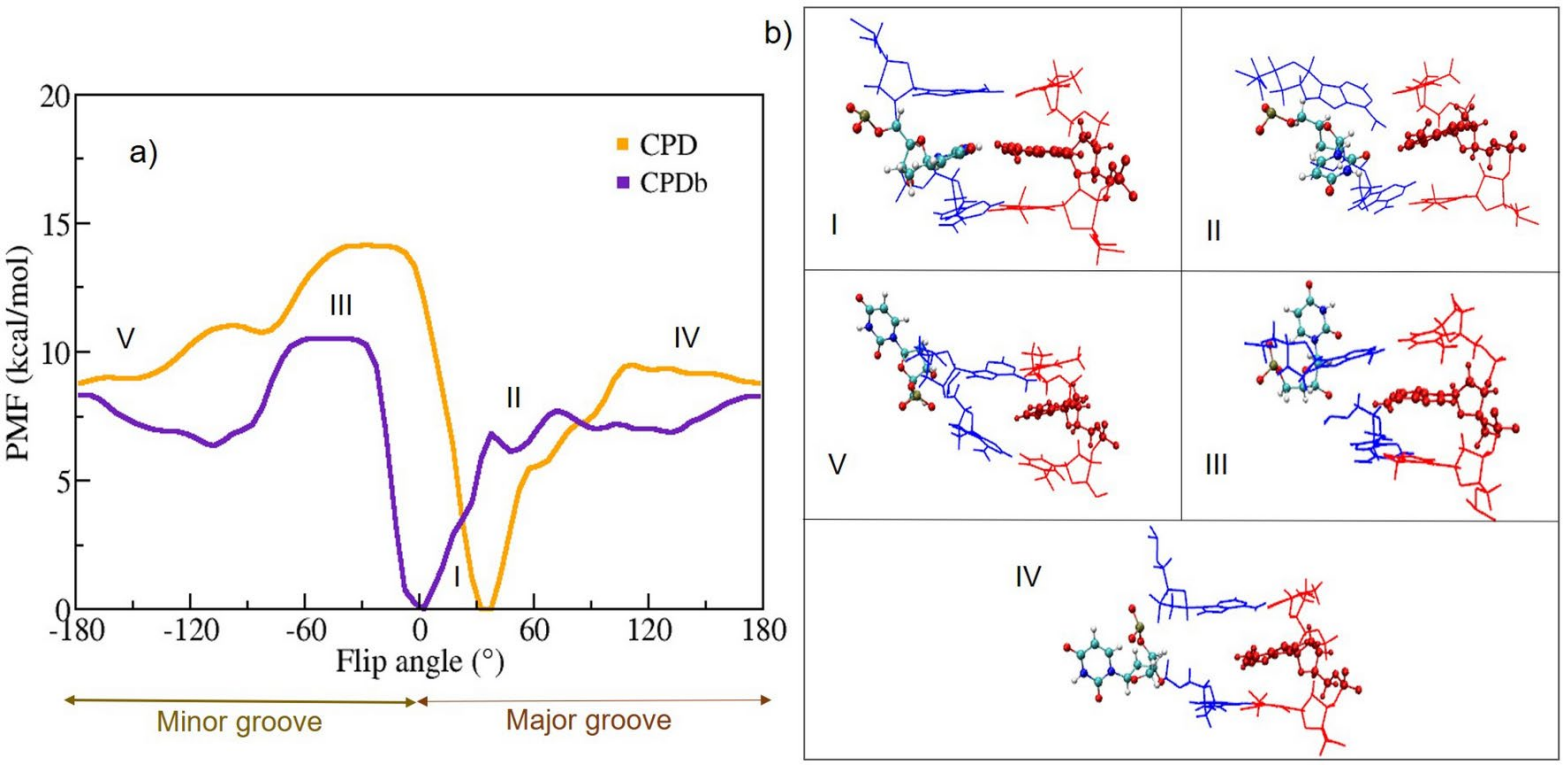
(**a**) Free energy profile (Potential of Mean Force, PMF) as a function of the Center Of Mass (COM) of the pseudo-dihedral angle for the uracil-mutated dsDNA; (**b**) The representative snapshots for the uracil base flipping steps [here region I is the Watson-Crick base pair, II, III, V regions are the intermediate steps, and IV is the fully flipped state of uracil].

During the base flipping simulation, an external history-dependent bias potential is applied to the system which can be expressed as a sum of Gaussians along the collective variables (CV) to enhance the sampling efficiency. The CVs for the uracil base flip have been chosen following existing literature, *i.e.* different versions of the center-of-mass pseudo-dihedral angle (CPD), see^40^ and CPDa/b^31^. For further details, see “Methods”.

The metadynamics simulations were conducted for the uracil mutated dsDNA system for about 100 ns. The pseudo-dihedral angles (CPD and CPDb) were used as the coordinates for free energy mapping to describe the uracil flipped-in and flipped-out states using the potential of mean force (PMF) analysis as shown in Fig. 2a). The base opening pathway snapshots are shown in Fig. 2b). In this Figure, notable similarities and differences between the two energy profile schemes are observed. The global minima of uracil embedded states at approximately $10^{\circ }$ in CPDb correlate well with previous theoretical studies^41^, whereas in the CPD scheme, it is approximately $40^{\circ }$. Here, the preselected CPD reaction coordinates scheme yields slightly different results. Both PMF profile schemes display a separation into two distinct regions: the major groove and the minor groove. A nucleobase within the dsDNA aligns with CPDb $> 0^{\circ }$ when flipping into the major groove pathway, whereas it aligns with CPDb $< 0^{\circ }$ when flipping into the minor groove pathway. In Fig. 2a), the spontaneous flipping of uracil through the major groove has a lower free energy barrier than through the minor groove in both CPDb and CPD schemes, respectively. The lower free energy barriers and their corresponding base opening angles were about 7.5 kcal/mol and $30^{\circ }$ for CPDb and 6 kcal/mol and $60^{\circ }$ for CPD schemes, respectively. This observation is in good agreement with the previous meta-eABF simulation^41^. The flipped-out pseudo dihedral angle uracil was about $180^{\circ }$, and the corresponding free energy barriers were about 8.2 kcal/mol and 9 kcal/mol for CPDb and CPD schemes, respectively. The transition from the flipped-in to the flipped-out state of uracil has a higher free energy barrier in the CPD than in the CPDb schemes. The obtained results suggest that the observed differences could be attributed to the effect of the reaction coordinate definition on the details of the PMF. Furthermore, the results indicate that uracil flips out more frequently through the major groove pathway than through the minor groove pathway.

**Fig. 3:**
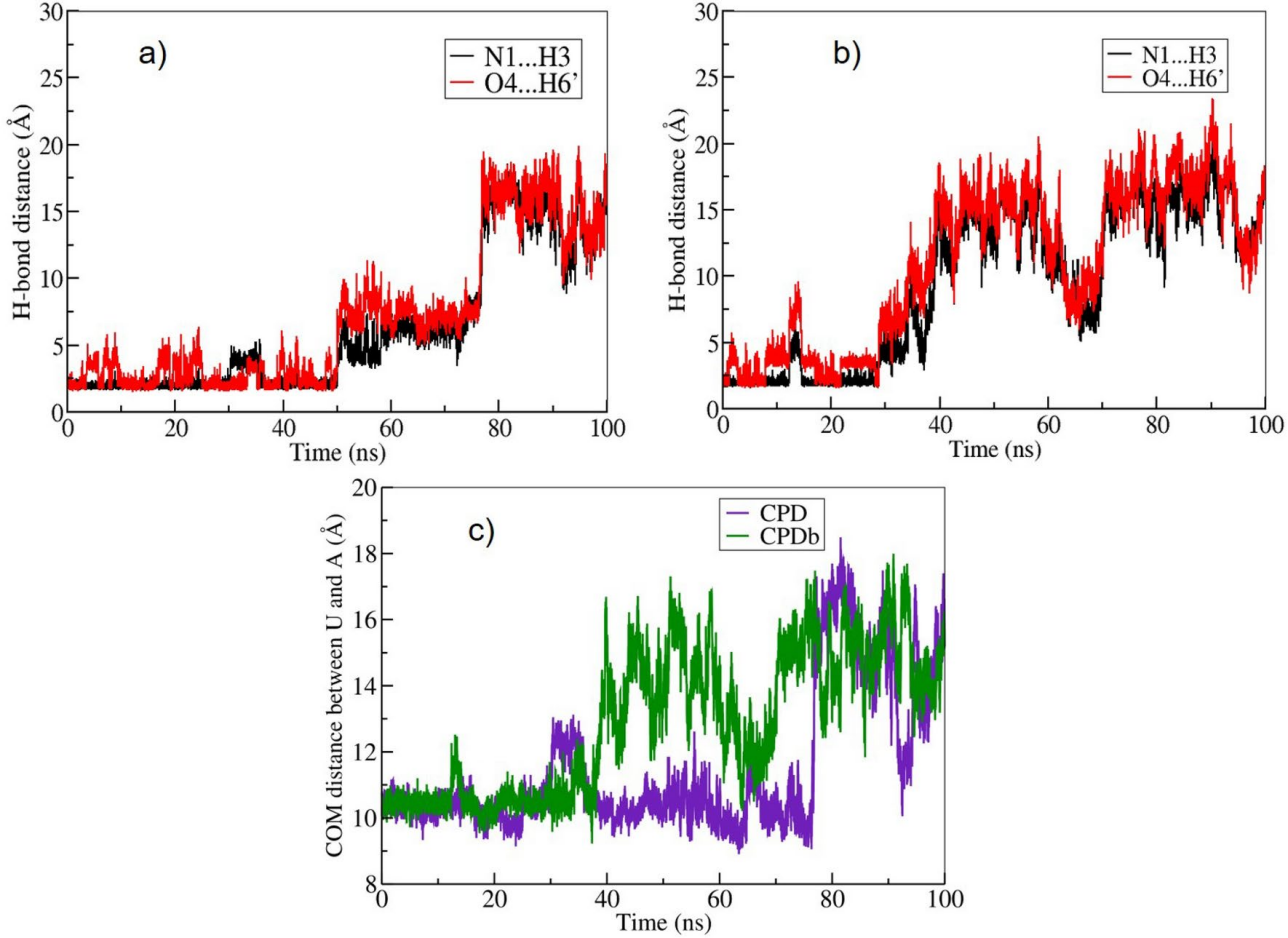
(**a**) and (**b**) the hydrogen bond distances between N1⋯H3 and O4⋯H6’ for the CPD and CPDb schemes during the course of the simulation, respectively; (**c**) Likewise, the calculated average centre of mass of the distance between the uracil and adenine residues for CPD and CPDb schemes.

In order to understand the structural properties of uracil base opening, analyses of the hydrogen bond distances (N1⋯H3 and O4⋯H6’) and the center of mass (COM) of distance between uracil and adenine were performed for the metadynamics simulation trajectories, as displayed in Fig. 3.

At the beginning of the simulation, the COM distance between uracil and adenine bases were about 10.5 Å. During the simulation between 30-40 ns, this distance increases slightly to 12.5 Å. After 75 ns (Fig. 3c), the uracil base is completely flipped-out, causing the distance to increase to 17 Å, whereas the hydrogen bond (H-bond) distance between N1(A)$\cdots$ H3(U) and O4(U)$\cdots$H6’(A) increased from 1.82 Å to 15.9 Å and from 2.12 Å to 18.4 Å, respectively; see Fig. 3a). In the CPDb (Fig. 3b) scheme, the COM distance increases from 10.5 to 15 Å, whereas the distances of the H-bonds N1$\cdots$H3 and O4$\cdots$H6’ have increased from 1.82 Å to 16.2 Å and from 2.08 Å to 16.4 Å, respectively.

The RDF analysis shows that, in both CPD and CPDb trajectories, a water molecule closely approaches the N6-H6...O4 bond between the uracil and adenine bases at an approximate distance of 1.9 Åduring the initial simulation time. In contrast, no water molecules are found near the N3-H3…N1 bond between the uracil and adenine bases (Supplementary Fig. S2). The RDF probability values of N6-H6...O4 for CPD and CPDb are approximately 0.2, which is further verified by the Independent Gradient Model (IGM)^42^, which provides insight into non-covalent interactions (NCI) between the molecules. During the initial stages of the simulation (0-30 ns), a transient water molecule approaches the O4 atom of the uracil base at a distance of  1.9 Å, forming a weak interaction (shown in Supplementary Fig. S3). The corresponding IGM isosurface value is 0.035, indicating a very weak interaction. Notably, this weak interaction does not influence the disruption of the N6-H6...O4 bond. Therefore, solvent effects do not play a important role in uracil base flipping under the studied conditions.

**Fig. 4:**
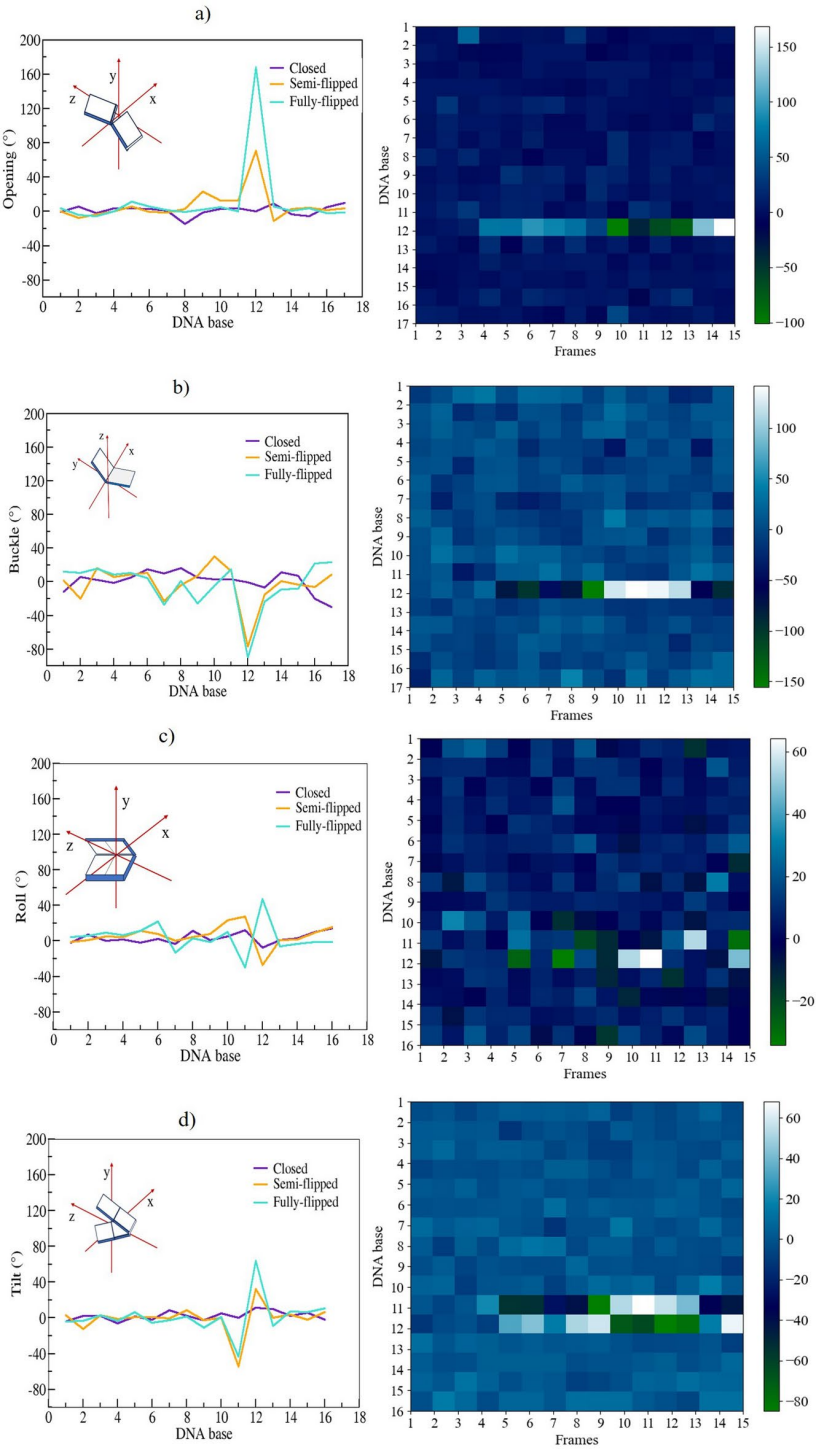
Intra-base pair parameters: (**a**) Opening and (**b**) Buckle, and inter-base pair parameters (**c**) Roll and (**d**) Tilt of uracil-mutated dsDNA for the CPDb scheme were calculated using the CURVES+ program. The plots on the right hand side are the respective heat maps of Opening, Buckle, Roll, and Tilt with respect to the trajectory frames. Here, the dark shades (blue to green) indicate negative values, and the light shades (cyan to white) indicate positive values of these quantities for the uracil-mutated dsDNA.

Further, we have analysed the uracil-mutated dsDNA structural deformations. For this we used the Curves+ program^43^. The results are given in Fig. 4 and the Supplementary Fig. S4. The intrabase parameters (buckle, opening) and the interbase parameter (tilt) for the flipped-in to flipped-out transition of uracil in the U.A base pair ranged from $-2.5^{\circ }$ to $-57.4^{\circ }$, $10.7^{\circ }$ to $168.2^{\circ }$, and $-1.6^{\circ }$ to $16.0^{\circ }$, respectively. The total dsDNA bending angle increased from $3.6^{\circ }$ to $68.6^{\circ }$. In particular, the major and minor groove values of flipped-out uracil dsDNA were about 11 Å and 7 Å, respectively. Since a wider major groove was confirmed more favourable for base flipping, the observed increase in the major groove width favors a structural adaptation that facilitates uracil flipping in the DNA sequence^37^.

**Fig. 5:**
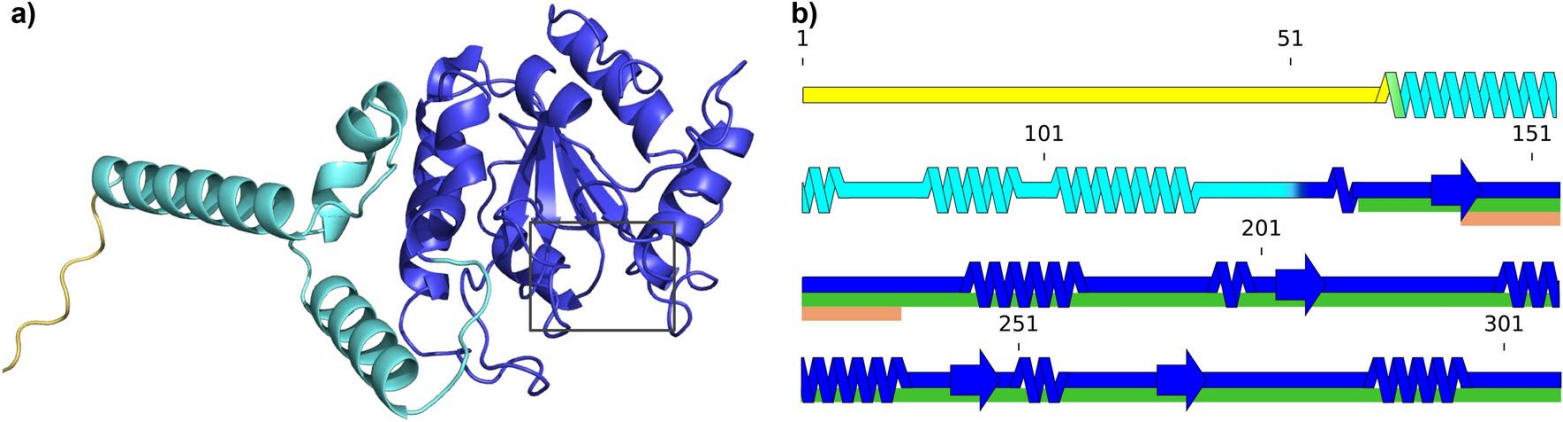
(**a**) Cartoon representation of UDG tertiary structure predicted with Alphafold2 (AF2) as described in “Methods”. The structure is represented with PyMOL^44^. Its residues are colored according to the backbone qualitative flexibility evaluation described in the main text and in “Methods”. The three flexibility levels are: *yellow* for flexible, *blue* for rigid and *cyan* for intermediate level between flexible and rigid. In (**a**), the black frame at the bottom right designates UDG’s catalytic pocket; (**b**) 2D representation of UDG secondary structure with the same color code as in (**a**), drawn with SSDraw^45^. Notable regions are highlighted under the concerned residues: the catalytic site is shown in *orange* (InterPro IPR018085) and the conserved UDG domain in *green* (InterPro IPR005122).

### Docking UDG to flipped uracil bases in dsDNA

Two isoforms of human UDG are known to originate from the alternative splicing of the UNG gene. The canonical isoform is UNG2 (Uniprot P13051-1) and has a length of 313 aa. It is expressed in the mitochondrion and is the one referred in the introduction as the first glycosylase characterized in complex with damaged DNA. The second isoform is UNG1 (Uniprot P13051-2, 304 aa), which is expressed in the nucleus and hence the isoform we used for our docking simulations. The difference between these two isoforms lies in their N-terminal beginning portions: UNG1 from residue 36 to its C-terminal end (304) is identical to UNG2 from residue 45 to its C-terminal end (residue 313). The structure of the predicted UNG1 structure we use is shown in Fig. 5a).

**Fig. 6:**
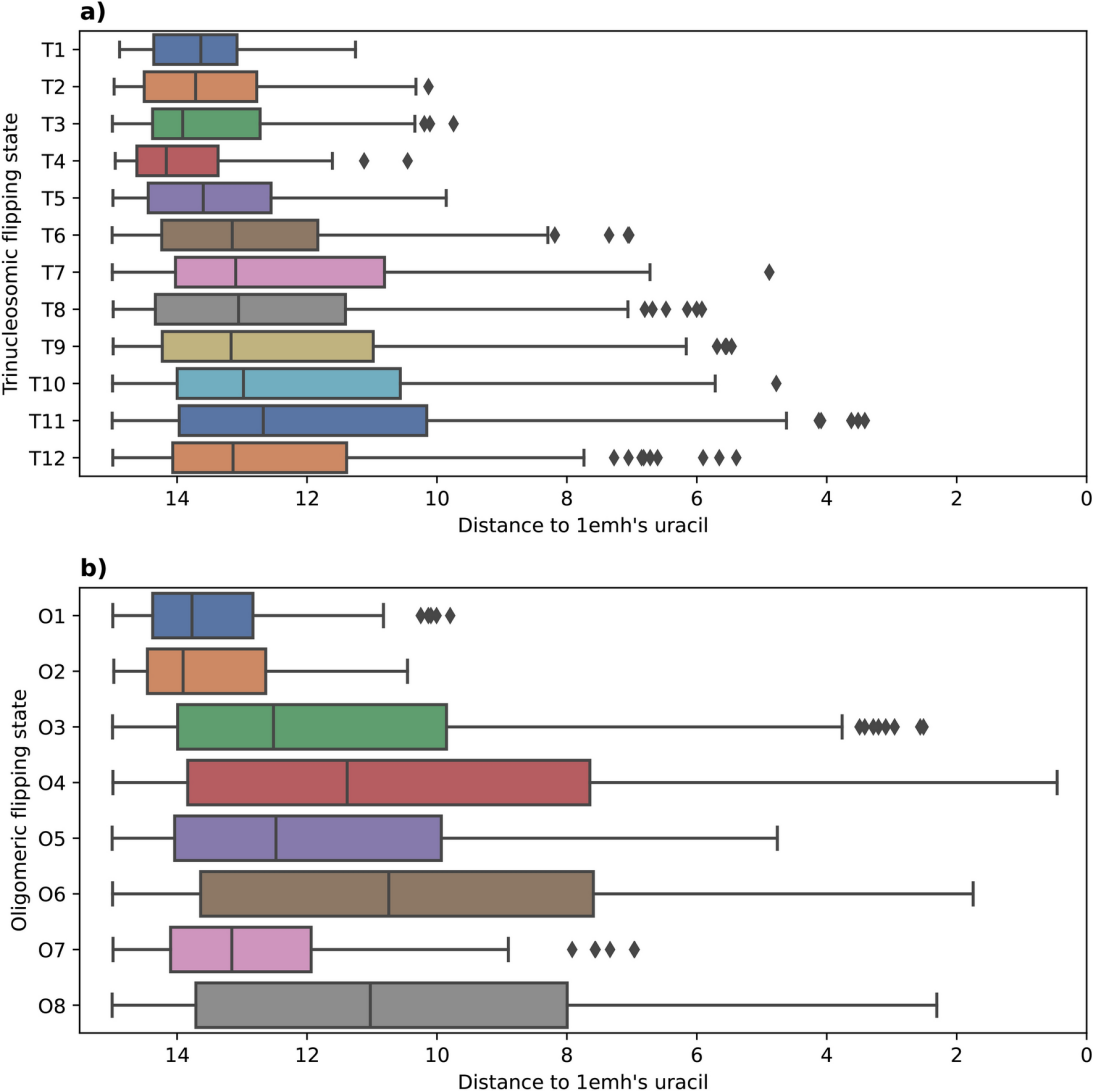
Distance of the docking-predicted uracil relative to the one of PDB ID: 1EMH, depending on the DNA structure used for docking with trinucleosomic strands in (**a**) and oligomeric strands in (**b**). The distance (Å) is computed after superimposing both UDG from the docked structure and from PDB ID: 1EMH. Only distances under 15 Å are conserved. X-axis goes from 15 Å to 0 Å. Distances are computed as described in “Methods” and illustrated in Supplementary Fig. S8.

As a first step in studying the docking process we have investigated the flexibility of UDG, since the docking of proteins to DNA is clearly influenced by the flexibility of both docking partners. The results of our investigation are reported in Fig. 5b). The flexibility information obtained with the MEDUSA webserver^46^(see “Methods”) reveals that the portion containing UDG’s catalytic site is predicted as mostly rigid. Upstream of this rigid section the protein is less rigid, even ending on a disordered and highly flexible N-terminal tail.

To study protein-DNA interactions, several docking algorithms and programs have been developed and are mostly available either from a webserver or with its code made publicly available, see “Methods”^47–52^. We have tested these docking programs on the crystal structure PDB:1EMH which we take as our reference structure for the recognition complex. The results of these docking attempts are summarized in Supplementary Fig. S6. We found that pyDockDNA performed best because it manages to discriminate between uracil and thymine, and was therefore selected for our docking simulations (see “Methods”).

In order to have a global view of UDG’s encounter with the damaged DNA strand, we took snapshots from the MD simulations of either the trinucleosome structure, as obtained in our previous work^26^, or from the MD base-flipping simulations described above. We denote these structures by either a ‘T’ for trinucleosome or ‘O’ for the oligomer (17-mer) dsDNA in the following. Table 1 collects the results of the measurement of suitable angles and distances to characterize the base-flip structures selected for the docking process. We characterized the docked structure by a distance and an angle measurement which is described in “Methods”.

**Table 1:** Quantitative description of impacting features for the structures ‘T’ from the trinucleosome simulations and ‘O’ from the 17-mer dsDNA oligomers, resulting from a gradual base flipping with their docking success evaluation. The distance $d_{U-groove}$ measurements and *Dihedral angle* metrics are described in Supplementary Fig. S5. The angle evaluates the extent of the base flipping, the distance indicates the minimal distance between the base and both edges of the groove. The closer the base is from the backbone of the opposite strand, the more sterically hindered it is. The sign “−” in dihedral angles implicates flipping towards the minor-groove.

	PDB ID:1EMH	T1	T2	T3	T4	T5	T6	T7	T8	T9	T10
Dihedral angle ($^\circ$)	− 171.75	−5.85	2.61	−4.71	−3.16	−8.66	−66.12	−96.58	−83.18	−108.82	145.12
$d_{U-groove}$ (Å)	20.8	11.8	12.9	14.2	11.5	12.9	12.0	10.4	10.8	11.1	8.3
Success	**High**	None	None	None	None	None	Low	Medium	Medium	Medium	Medium

	T11	T12	O1	O2	O3	O4	O5	O6	O7	O8	
Dihedral angle ($^\circ$)	−119.20	175.9	41.8	29.8	151.9	−173.2	−103.6	151.7	103.9	160.5	
$d_{U-groove}$ (Å)	12.8	10.4	14.4	15.2	13.2	16.4	10.9	15.8	10.0	13.3	
Success	Medium	Medium	None	None	**High**	**High**	Medium	**High**	Low	**High**	

A common feature appearing for every successful docking, as shown in Table 1, is a sufficiently wide opening angle of the flipped-out uracil base and a reasonable distance measure (the lesser being 13 Å) to the opposite DNA strand’s backbone. Both conditions are to be fulfilled for UDG to have enough space to reach the damaged base. These two conditions appear to be the main factors for a high success in docking as compared to the less successful conformations.

To analyse the docking results, we first filtered the conformations sampled by pyDockDNA. From the initial 10,000 docked structures that pyDockDNA produces with FTDOCK, it selects the 100 best according to a scoring function, which in this case is the one with no desolvation. According to the Supplementary Fig. S7a), this associated score is not sufficient to differentiate between the good and the bad docking solutions.

In order to sort the predictions by relevance, i.e. by how well the obtained interface corresponds to the one observed in PDB ID:1EMH, we went back to the initial 10,000 sampled conformations and devised a protocol described in “Methods” which measures the similarity of the uracil position between the docking structure and PDB ID:1EMH used as our reference structure. This measure, represented in Fig. 6 is then translated to an indicator listed in Table 1 as ‘success’. According to Fig. 6, the DNA strand with the highest success with a near perfect similarity to PDB ID:1EMH is ‘O4’. Its docking success coincide with the structure having the highest value in both the opening angle and the groove width according to Table 1.

**Fig. 7:**
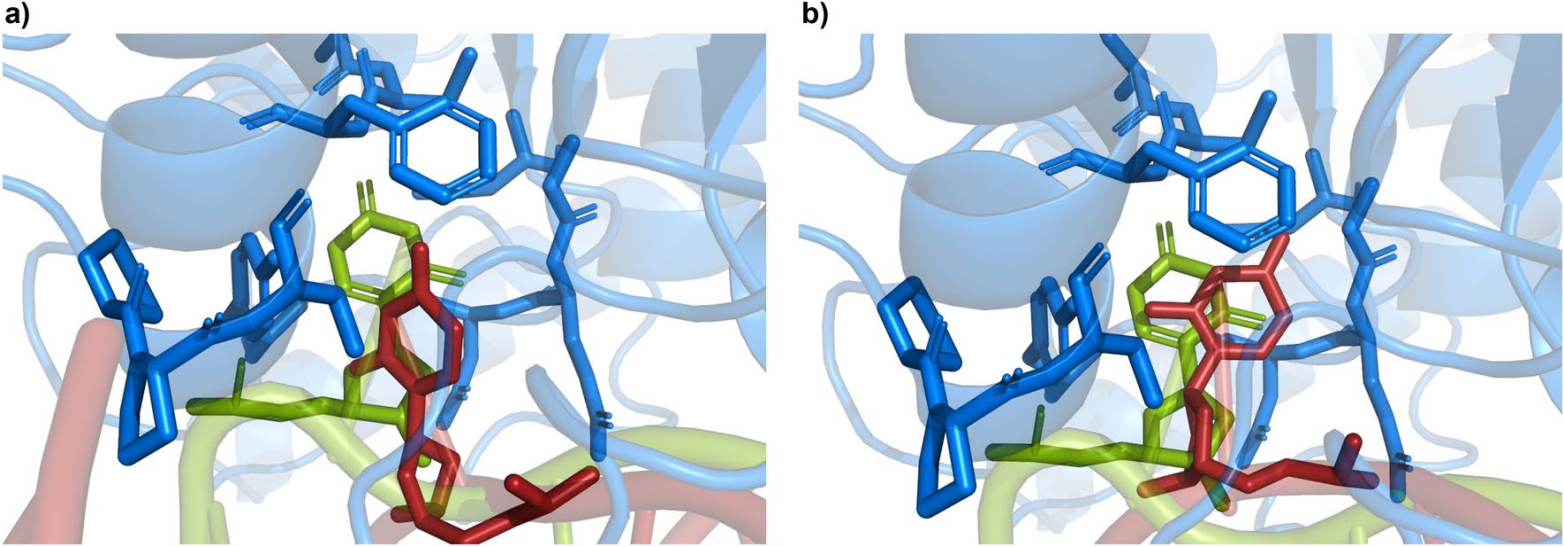
Docked structure for the highest success of both types of induced base flipping (‘T’ and ‘O’), identified according to Fig. 6. (**a**) ‘T11’ DNA strand (red) docked to UDG (blue; cartoon) with a focus on its catalytic pocket (blue; sticks). The uracil (red; sticks) from the docked DNA is compared to the one from PDB ID:1EMH (green; sticks) as the reference. Following the same color code, (**b**) shows the ‘O4’ DNA strand docked to UDG with PDB ID:1EMH uracil as a reference to assess how well docking predicts the entrance of uracil into the catalytic site of UDG.

According to these measures, the highest docking success of both ‘T’ dsDNA and ‘O’ dsDNA (see Fig. 6), respectively ‘T11’ and ‘O4’, were selected for illustration purposes in Fig. 7. It denotes the high similarity of these docking successes to the PDB ID:1EMH structure. ‘T12’ has a way wider flipping angle while being less successful than ‘T11’ (Fig. 6a) for docking to UDG. The determining factor making ‘T11’ the most prone among the ‘T’ dsDNA to interacting with UDG seems to be the large distance between its uracil and the nearest groove’s end. On the other hand, ‘O4’ has at the same time the widest angle of base flipping and the most space in the groove making it the top pick for a successful docking.

**Fig. 8:**
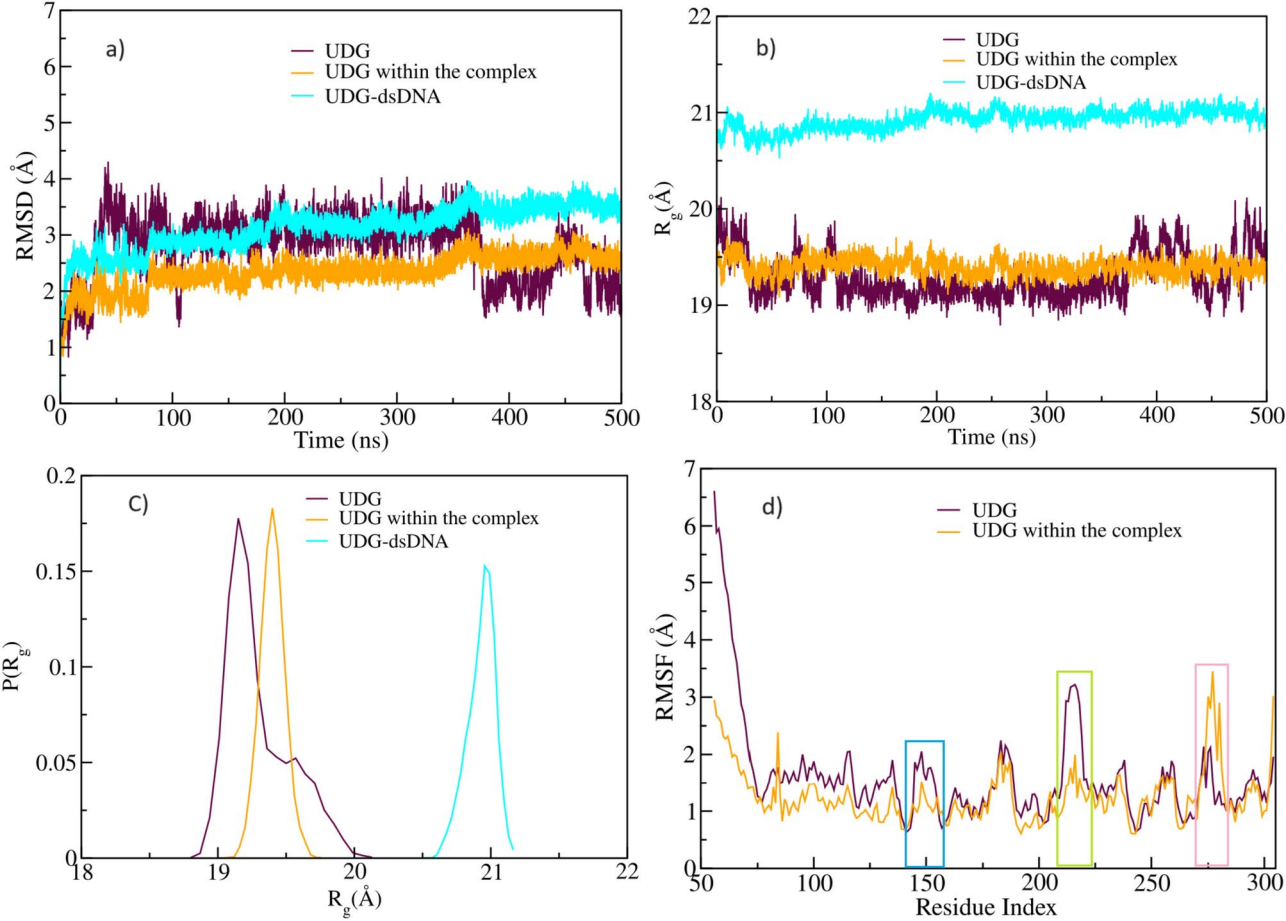
Structural analyses for the UDG-dsDNA complex. (**a**) Root mean square deviation (RMSD), (**b**) radius of gyration ($R_g$), (**c**) probability distribution of $R_g$, and (**d**) root mean square fluctuation (RMSF). Here, the blue box highlights the catalytic site of the UDG enzyme, the green box indicates the fluctuation of loop residues (residues 210 to 220), and the pink box indicates the fluctuation of LEU272 intercalated into the dsDNA within the UDG-dsDNA complex.

### Structural dynamics of uracil-flipped dsDNA bound to UDG enzyme

The optimal docking conformation of uracil-flipped dsDNA with the UDG enzyme (structure ‘O4’) was further explored using all-atom molecular dynamics simulations for 500 ns. The superposition of the docked complex and the final snaphot of the MD simulation are shown in Supplementary Fig. S9. The overall structural stability, compactness, and the flexibility of the complex system were evaluated using RMSD, radius of gyration ($R_g$), and RMSF analyses. The mean RMSD values of the protein, the protein within the complex, and the protein-dsDNA complex were computed as 2.77 Å, 2.35 Å, and 3.14 Å, respectively, as shown in the Fig. 8a). The compactness of all three systems was assessed using $R_g$ and the probability distribution of $R_g$, as illustrated in the Fig. 8b and c. UDG initially exhibited higher fluctuations, around 20 Å, between 0 and 100 ns. After 100 ns, these fluctuations began to decrease to approximately 19.5 Å. However, after 350 ns, the UDG experienced increased fluctuations, primarily due to the loop present in the catalytic site (residues 210-220), which became more flexible and fluctuated significantly during the simulation. Consequently, the probability distribution of $R_g$ adopted a bimodal conformation. In contrast, the $R_g$ values for the UDG within the complex and the UDG-dsDNA system remained stable throughout the simulation, with values of 19.4 Å and 21 Å, respectively. The flexibility of the systems was further analysed using RMSF, as shown in the Fig. 8d. The flexibility of the loop present in UDG is higher than that in the complex system, as shown in the Fig. 8d. Notably, the loop residues (residues 145-158) located at the catalytic sites exhibit slight flexibility, with an RMSF of approximately 2.4 Å. The presence of uracil at the catalytic sites in the complex stabilizes the loop, rendering it more rigid due to the formation of hydrogen bonds with GLN144 (URA(O4)⋯H), ASP145 (URA(H5)⋯O), and PRO167 (URA(O2)⋯H) which are shown in Supplementary Fig. S10. The loop residues 210-220 in the protein system are highly flexible, with an RMSF range of approximately 3.0-3.8 Å. These residues are further stabilized by the dsDNA during complex formation. In the complex, amino acids 272-278 exhibit higher fluctuations because the LEU272 residue in the loop intercalates into the nucleic acid strand to fill the gap created by uracil flipping into the catalytic site of the UDG enzyme, which correlates well with previous experimental findings^2^. As for the initial stages of base flipping we do not find a relevant influence of the solvating waters on the binding of UDG to the flipped uracil base.

## Discussion

In this work we present arguments that the mechanism by which UDG identifies uracil bases in dsDNA cannot rely alone on a random search combined with the ‘pinch-push-pull’-model. We purport that Uracil bases can present themselves to UDG via stochastic, thermally-induced base-flipping, but even more readily due to the ubiquitous presence of forces acting on dsDNA. As we have shown earlier^26^, this leads to instabilities of the dsDNA in the course of which bases flip out and become accessible to UDG. Here, we have studied whether this scenario is in accord with the base-flipping process of uracil from dsDNA, and whether the resulting structures can be properly recognized by UDG, i.e. whether a recognition complex between uracil and UDG can be formed. Our approach consisted in studying the base-flipping of uracil from dsDNA oligomers, using a collective variable/metadynamics approach within molecular dynamics simulations. In these simulations, uracil-flipped states were generated, passing through different stages of the flipping process in time. In parallel, we have selected base-flipped structure from our earlier large-scale MD simulations of trinucleosome arrays, in which base-flipping has occurred in the linker DNA of the arrays in the course of a mechanical instability in the compressed dsDNA. The opening angles of all of these structures have been quantified.

Subsequently, we have performed a study of rigid docking of UDG to the base-flipped uracil in dsDNA. While several protein-DNA docking programs have been developed in recent years, the study of protein-DNA docking is generally much less developed than the methodologies for protein-protein docking. In addition, the case of base-flipping requires the docking to be performed not towards a pristine, but rather a dynamically perturbed structure. This requirement has both necessitated a detailed study of available protein-dsDNA docking softwares, as well as a careful investigation of the docking success, i.e. the formation of a ‘proper’ recognition complex. We found pyDockDNA to be the best suited software for our task. For the evaluation of the quality of the docking for our predicted recogition complexes, we took the crystal structure of UDG-dsDNA from PDB ID:1EMH as our reference structure. Our careful evaluation of the docking quality for both the oligomeric and trinucleosomic dsDNA is documented in Supplementary Fig. S6. Overall, we identify two crucial factors in obtaining UDG-dsDNA recognition complexes, both of which are related to the opening angle of the uracil base. Firstly, we find that only a nearly fully flipped base (meaning between an angle of 150° and 180°) allows for recognition complex formation. The second crucial factor is the increase in width of the groove into which the base opens. The latter fact is well-known from earlier experimental studies of uracil embedded in nucleosomal DNA^53^. If these conditions are met we find that the formation of a high-quality recognition complex according to our criteria, as given in “Methods”, is possible. This is supported by MD simulations we performed on the best obtained complex.

Therefore, our study provides evidence that, besides the binding of UDG to dsDNA and its possible participation in a ‘pinch-push-pull’ mechanism, mechanical instabilities of dsDNA in the crowded nuclear environment that favor a destabilization of the dsDNA, and therefore enhance the probability of base flipping beyond a merely random, thermally assisted event, may represent a relevant, alternative or complementary pathway allowing the identification of uracil by UDG, thereby initializing the base-excision process. Our finding is further supported by the discussion of the effect of DNA deformation on protein-DNA complex formation in^25^.

The initial BER recognition process is in fact occurring in a highly dynamic environment and very closely linked to DNA dynamics, at least in chromatin linker DNA. In future work we intend to look at the role of DNA dynamics in nucleosomes on the BER process.

## Methods

### Selecting and preparing the MD-snapshots ‘T’ from the trinucleosome simulations

We have selected twelve snapshots from the trinucleosome simulations described in^26^. They were chosen from the structure called T183, which was reconstructed by assembling three copies of the 197 nucleosome (PDB file 5NL0)^54^. More details as well as access to the trinucleosome structure can be found in^26^. The sequence of the dsDNA sequence extracted from that structure is given by 5’−TACGUATGG−3’. In the original MD-simulations a fully paired dsDNA structure was used. The mechanically flipped-out base was replaced by uracil using the ’Mutation’ section in the w3DNA 2.0 webserver^55^.

### MD simulations of uracil flipping in the dsDNA oligomer ‘O’

All atom molecular dynamics (MD) simulations were executed with the NAMD 2.14 package^56^ with the CHARMM 36 force fields^57^. A concentration of 0.15 mol/L $\text {Na}^+$ and $\text {Cl}^-$ ions were added at random positions to maintain the charge neutrality of the dsDNA system. The all-atom simulations employed periodic boundary conditions (PBC) and multiple time-stepping wherein local interactions were considered every 2 fs and full electrostatic evaluations were conducted every 2 time steps. The particle mesh Ewald method (PME) was employed for long-range electrostatic calculations^58^. The simulation box size was $79.7 \times 40.6 \times 40.9$ Å$^3$ with a distance of 10 Å  between the box edges and the dsDNA. The cutoff and switching distances were set at 12 Å  and 10 Å , respectively. Covalent bonds involving hydrogen were held rigid by the RATTLE^59^ and SETTLE algorithms^60^. The dsDNA system was minimized for 5000 steps using the conjugate gradient method. The pressure (constant NPT) was maintained at 1 atm, and the temperature was kept at 310 K. Temperature control was attained through Langevin dynamics for all non-hydrogen atoms, while pressure was controlled using a Nose-Hoover Langevin piston. The trajectories of the simulations were visualized and analysed by the visual molecular dynamic program (VMD 1.9.4)^61^ and the dsDNA conformational analysis were calculated by Curves+ program^43^.

### Choice of collective coordinates

In this study, the reaction coordinates have been described by three choices i) the centre-of-mass (COM) of pseudo-dihedral angle between the flipping base, the sugar group of the same nucleotide (group 1), the sugar group of the next nucleotide (group 2), the base of the next nucleotide plus the base of the opposing nucleotide (group 3), and the target base (group 4); ii) the center of the two flanking base pairs (group1), group 2 and 3 are defined by the flanking sugar moieties, and group 4 is defined by the target base for the flipping process; and iii) in the CPDb scheme, the group 1 is same as in CPDa, group 2 and 3 are defined by the flanking phosphate group, and the group 4 is defined by the target base for the flipping process. Among these choices, the uracil base opening event did not ensue with the CPDa scheme. As a result, all calculations described here were conducted using the CPD and CPDb schemes. The potential mean force was calculated for reaction coordinate between − 180° and 180° into windows of $5^\circ$ degree width; settings for Gaussian hillWeight = 0.001 kcal/mol and hillWidth = 2 bin width were added in the metadynamics simulation.

### Definition of docking partners

Docking here consists in simulating the interaction between UDG and the base-flipped DNA. The DNA strands used in these simulations are from the previous step of producing ‘O’ dsDNA and snapshot selection for ‘T’ dsDNA described above. As for ‘O’ dsDNA, over the 14,000 frames of the simulations, we selected the 8 following frames, ranging that we call O1 to O8, respectively: 6983, 7269, 7712, 8272, 9040, 10145, 10394, 11433. For UDG, the structure has been predicted by AlphaFold2 (AF2) with the isoform sequence from Uniprot ID P13051-2. The 56 first residues were discarded as they are in a disordered region with no confidence in its position. This structure is represented in Fig. 5 with PyMOL^44^.

### Measurements of angles and distances in the base-flipped conformations

The opening angles for the flipped-out uracil bases in Table 1 are determined via the collective variable approach, as defined in the text. The method has been used for both the trinucleosome (‘T’) and control (‘C’) structures. The $d_{U-groove}$ measure is calculated between the flipping out uracil and the nearest groove’s extremity. This distance spans between the N1 uracil atom and the nearest phosphate from the opposite strand. For an illustration, see Supplementary Fig. 3a, in which four positions P1, P2, P3, and P4 are defined representing the pseudo-dihedral points used to measure the uracil flipping angle around its backbone during the simulation.

### UDG flexibility

Flexibility was predicted using the MEDUSA webserver^46^, https://www.dsimb.inserm.fr/MEDUSA/. It predicts flexibility from the protein amino acids sequence in different modes. Each of these modes assign a probability of belonging to a predefined number of flexibility class per residue. The mode we have used is 3 class prediction; the results are summarized in Fig. 5a and b. The structure in Fig. 5a is represented with PyMOL^44^, Fig. 5b is drawn with SSDraw^45^. The flexibility classes assigned according to MEDUSA are used as coloring factors; the explanation is given in the Figure caption.

### Selection of protein-DNA docking software

In order to determine which was the best-suited available software for UDG-dsDNA docking, we have tested different types of docking software. The complete list is given by: HDOCK^49^, HADDOCK^62^, pyDockDNA^51^, MDockPP^63^, RoseTTAFold2NA^64^ and AlphaFold3^65^. They were all evaluated on reproducing the interaction in the 1EMH crystal structure. From our tests, pyDockDNA performed the best on our use case as it discriminates between Uracil and Thymine, thus being the one selected to run the docking simulations. The summary of the comparison is found in Supplementary Fig. S4.

### Evaluation of the docking structures

This protocol takes the PDB ID:1EMH structure as a reference for the highest docking quality; it is the target which we wanted to reproduce by docking, and putting it more precisely, we measured the similarity with which the uracil enters the UDG compared to PDB ID:1EMH. After aligning PDB ID:1EMH UDG and the UDG of every docking conformation for each DNA strand with different base flipping angle and protocol (10,000 docking poses per simulation), we measured the distance between the N3 atoms of the evaluated uracil (U1) and the uracil analog 2’-deoxypseudouridine of the reference uracil (U2). This $d_{U1-U2}$ distance (in Å) is a direct quantitative evaluation of how much the docking conformation corresponds to the experimental structure. Figure 6a and b both represent this evaluation performed on the trinucleosomic structure (‘T’ in a) and the oligomeric structure ‘O’ in b). The goal of this measure was to highlight which DNA strand features are impacting UDG action the most, thus the most successful docking conformation for each strand was taken and its quality was classified into ‘None’, ‘Low’, ‘Medium’ and ‘High’ quality according to the thresholds:

- ‘None’: $$d_{U1-U2} > 9$$ Å
- ‘Low’: $$9 \, {{{\AA }}}> d_{U1-U2} > 6$$ Å
- ‘Medium’: $$6 \,{{{\AA }}}> d_{U1-U2} > 3$$ Å
- ‘High’: $$d_{U1-U2} < 3$$ Å

## Supplementary Information

Supplementary Information.

## Data Availability

All data and scripts of the article are hosted on this GIT repository: https://gitlab.in2p3.fr/cmsb-public/udg_dna.
