# Supplementary material for: The ‘very moment’ when UDG recognizes a flipped-out uracil base in dsDNA
Source: Sci Rep. 2025 Mar 7;15:7993. doi: 10.1038/s41598-025-91705-6 (PMC11889109; doi:10.1038/s41598-025-91705-6)
Supplement: Supplementary file 1 — Supplementary Information. [file 41598_2025_91705_MOESM1_ESM.pdf]

# Supplementary Material to The 'very moment' when UDG recognizes a flipped-out uracil base in dsDNA

**Vinnarasi Saravanan[1], Nessim Raouraoua[1], Guillaume Brysbaert[1], Stefano Giordano[2], Marc F. Lensink[1], Fabrizio Cleri[3], and Ralf Blossey[1,*]**

[1]University of Lille, CNRS, Unité de Glycobiologie Structurale et Fonctionnelle (UGSF), UMR8576, 59000 Lille, France
[2]University of Lille, CNRS, Centrale Lille, Univ. Polytechnique Hauts-de-France, UMR 8520 - IEMN - Institut d'Electronique, de Microélectronique et de Nanotechnologie, F-59000 Lille, France
[3]University of Lille, Institut d'Electronique, de Microélectronique et de Nanotechnologie (IEMN CNRS UMR8520) and Département de Physique, 59652 Villeneuve d'Ascq, France
[*]ralf.blossey@univ-lille.fr

## ABSTRACT

This Supplementary Material contains Supplementary Text and Figures to accompany the Main Text.

## An estimate of the efficiency of the uracil search process by UDG

About 200 to 500 C-to-U deaminations per cell per day are thought to occur naturally, both by exogenous and endogenous chemical oxidation events [S1]. We may consider that upon therapeutic irradiation such a number could increase even $\sim$ 100-fold, mostly by the action of free water-radicals. The average daily concentration of DNA-U in the typical cell nucleus ($\varnothing \sim 7$ mm, V $\sim$ 1.4 pL) can therefore expected to be about 500 to 50,000/(6,02 $\cdot 10^{23}$) = $8 \cdot 10^{-22}$ to $10^{-20}$ mol per 1.4 pL, that is 6 $\cdot 10^{-10}$ to 6 $\cdot 10^{-8}$ M.

UDG is found in the cell nucleus in concentrations that can vary between 0.1 to 10 ng/ml, according to the sensitivity range of ELISA lab assays. Hence, a typical nucleus should contain a few $10^{-16}$ grams of UDG, with a molar weight of 25,7 kDa, that is order of 2,000 to 100,000 copies of the protein, also fluctuating during the cell cycle phases [S2]. Such a concentration seems nicely adjusted to match a ratio of roughly 1 UDG per uracil/day. However, if the absolute concentration could be about right, the main question is how UDG can identify the defective base among the 6 billion nucleotide pairs, that is the kinetics and the success rate of the repair process.

Experiments can track the kinetics of association of UDG with properly prepared DNA samples. For example, Tainer et al. [S3] measured by stopped-flow electrophoresis the excision kinetics of UDG against small (19-nt), linear DNA fragments, either pristine, or including U:A and U:G mispairs. They stocked 8.5 pmol of DNA (that is, about 5 $\cdot 10^{12}$ copies of the oligonucleotide) and 0.02-0.04 ng of UDG (that is, about 5-10$\cdot 10^{8}$ copies) in a reaction volume of 40 $\mu$l. To compare with the in-vivo conditions, one may note that the DNA (besides being fragmented into tiny oligomers, and without histones) is diluted to about 2,000 times smaller than in the cell nucleus, whereas the UDG concentration is diluted to 2,500, so the relative proportions of UDG/DNA are roughly respected. However, the concentration of uracil is unrealistically high, since in the experiments there is one U every about 20 nt-pairs, although the DNA is broken and not continuous. On a proportional basis, this corresponds to about 1 UDG per 10,800 DNA-U defects, and is comparable to concentration ratios used in other similar experiments, see, e.g., [S3].

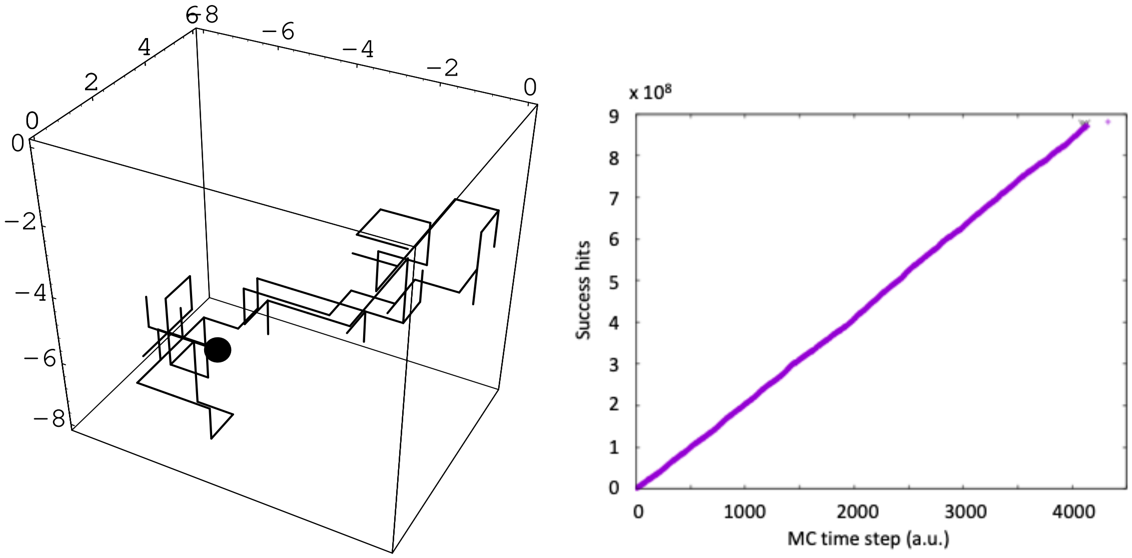


**Figure S1.** Figure 1 a) UDG performing a Brownian random walk; 1 b) Total number of success hits as a function of simulation time.

We ran a very simple Monte Carlo random search simulation, with the aim of estimating hitting times and search times of a single UDG along a fragment of DNA. We represented the search space as a dense collection of small cubic volumes of side 7 nm. Since the size of the globular UDG protein is roughly 5 nm, we imagine that each small cube is large enough to accommodate one copy of UDG and one fragment of DNA, in which case we count the couple as a successful identification of the uracil by UDG. Given the above concentration of 1 UDG per 10,800 DNA-U, it is enough to consider a space of $(650)^3$ = 2.75 $\cdot 10^8$ cubes, including 10 800 randomly placed DNA at fixed positions, and one UDG performing a Brownian random walk, as shown in Fig. S1 a). At each MC time step the UDG jumps from one cube to another adjacent cube, also including stepping back to the starting one. Whenever UDG finds a DNA-U in a volume, a successful hit is counted. The slope of the linear plot in

Fig. S1 b), displaying the total number of success hits as a function of the fictitious simulation time, gives a recursion time $\Delta$ t $\sim$ 25 000 Brownian steps, between two successive hits of UDG on two distinct DNA oligomers. The effective diffusion constant in water (with a kinetic viscosity $\eta = 9.3 \cdot 10^{-4}$ Pa s) of a globular protein with the size R = 2.5 nm of UDG, can be estimated by the Einstein relation

$$D = \frac{k_B T}{6\pi\eta R} = \frac{4 \cdot 10^{-21}}{6\pi \cdot 9.3 \cdot 10^{-4} \cdot 2.5 \cdot 10^{-9}} \frac{m^2}{s} = 9 \cdot 10^{-11} \frac{m^2}{s}$$

from which the elementary diffusion time per each step of 7 nm between two cubes, is estimated as $\tau \approx 0.54\,\mu s$. Hence, a $\Delta t \sim 25000\,\tau \sim 13.5$ ms is the average time between each two successful hits on two distinct DNA oligomers, at the experimental concentrations.

By looking at the linear part of the kinetic plot reported in Tainer's paper ([S3], Figure 1) a time of about $\sim$ 5 minutes is required to excise $\sim$ 180 $\mu$mol U per mg of UDG, or 4,600 Uracil per UDG, that is about 65 ms for each event. The difference of about 50 ms between our calculated hitting time of 13,5 ms, and the excision time of 65 ms, appears to support the model put forward by the authors, according to which the UDG must firstly attach at a random location along the DNA oligomer, and then spends time to migrate along the length, "searching" for the defective nucleotide pair.

However, while such a picture seems to hold well for the experimental situation in which just 10-20 bases have to be searched in each fragment, it turns to fully unrealistic when translated to the in-vivo situation. Even neglecting the presence of densely packed chromatin into millions of nucleosomes, and taking the DNA as a free long, continuous polymer, at 10- to 100,000 uracil per 6 billion nt-pairs, each UDG should scan millions of nucleotide pairs according to this model: if 50 ms are needed to scan just about 20 nt-pairs in the experiment, finding one uracil by similarly scanning several million nt-pairs would require a time of days.


[S1] Lindahl, T., Instability and decay of the primary structure of DNA, Nature **362**, 709–715 (1993)
https://doi.org/10.1038/362709a0

[S2] Fischer, J.A. and Caradonna, S.J., Analysis of Nuclear Uracil DNA–Glycosylase (nUDG) Turnover During the Cell Cycle, in: Cell Cycle Synchronization, Methods in Molecular Biology (MIMB, volume 1524), 177-188 (2016)
https://doi.org/10.1007/978-1-4939-6603-5_11

[S3] Parikh, S.S. et al. Base excision repair initiation revealed by crystal structures and binding kinetics of human uracil-DNA glycosylase with DNA, The EMBO Journal **17**, 5214-5226 (1998) https://doi.org/10.1093/emboj/17.17.5214

[S4] Bellamy, S.R.W. and Baldwin, G.S., A kinetic analysis of substrate recognition by uracil-DNA glycosylase from herpes simplex virus type 1, Nucleic Acids Research **29**, 3857–3863 (2001) https://doi.org/10.1093/nar/29.18.3857

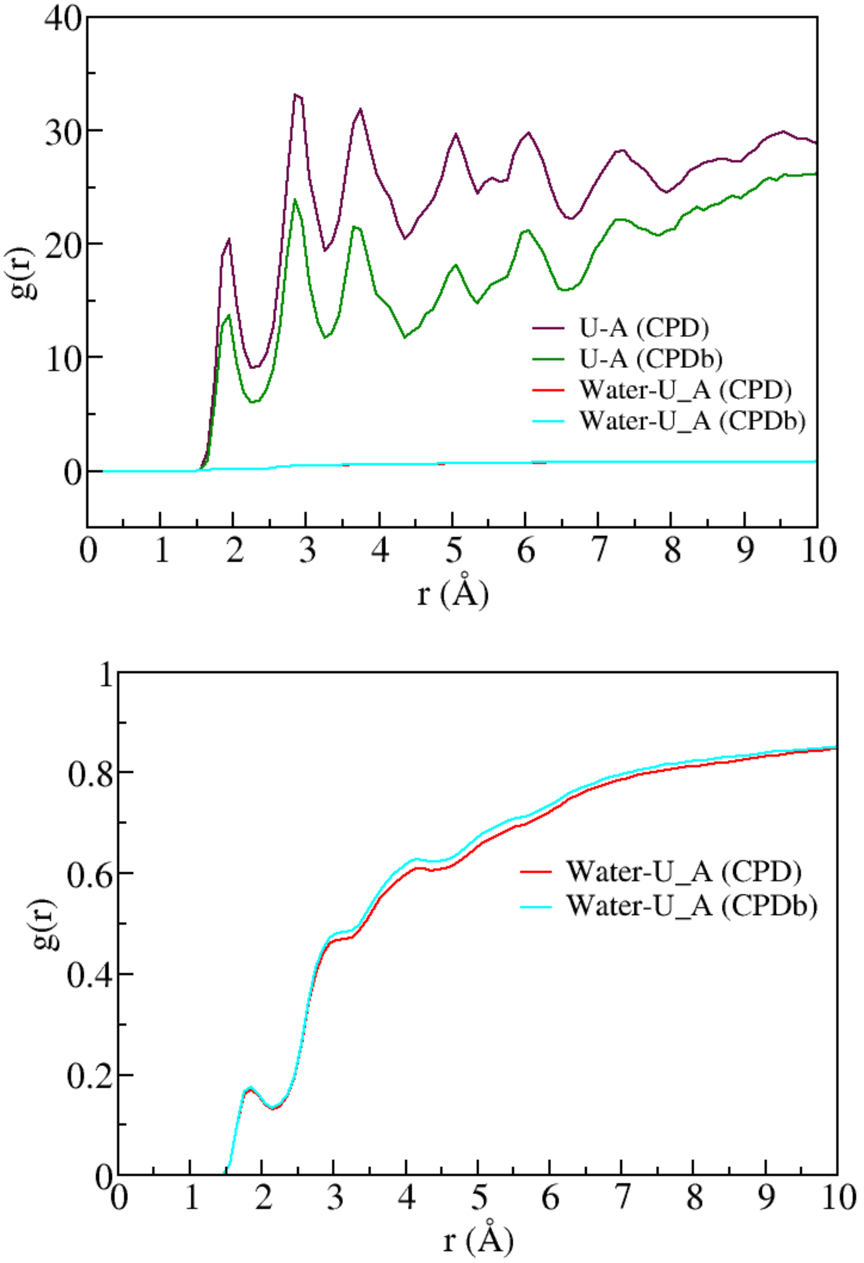


**Figure S2.** The radial distribution function (RDF) calculated for CPD and CPDb trajectories to analyse the role of water molecules in uracil base flipping in dsDNA. Top: comparison of the RDF for uracil-adenine and nearby water molecules; bottom: water molecules near the uracil and adenine bases.

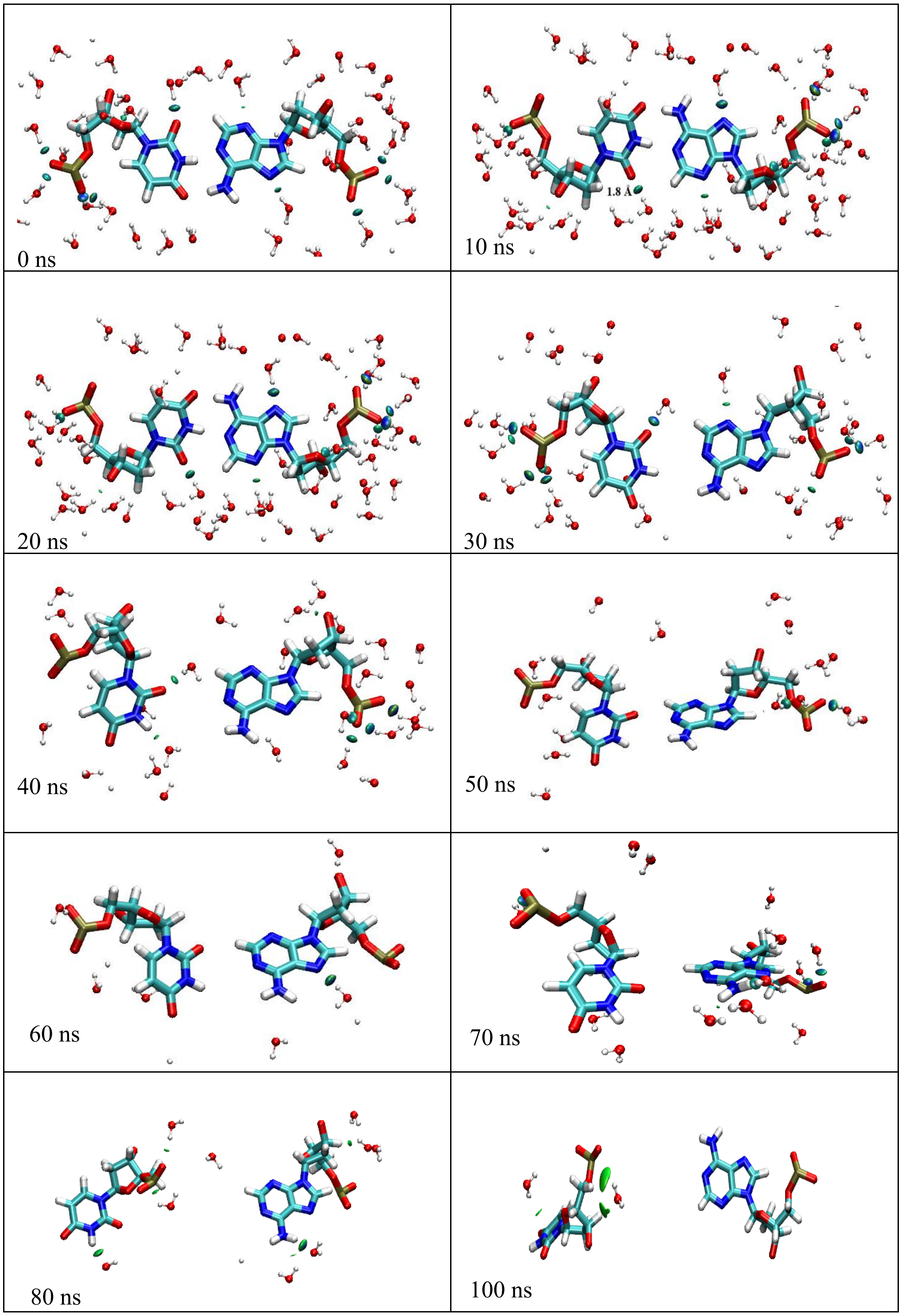


**Figure S3.** The Independent Gradient Model (IGM) calculation for the uracil-adenine base pair with surrounding water molecules within 5 Å, used to identify molecular interactions between the bases. The green-coloured isosurface represents non-covalent interactions between bases and the water molecules, with a value of 0.035.

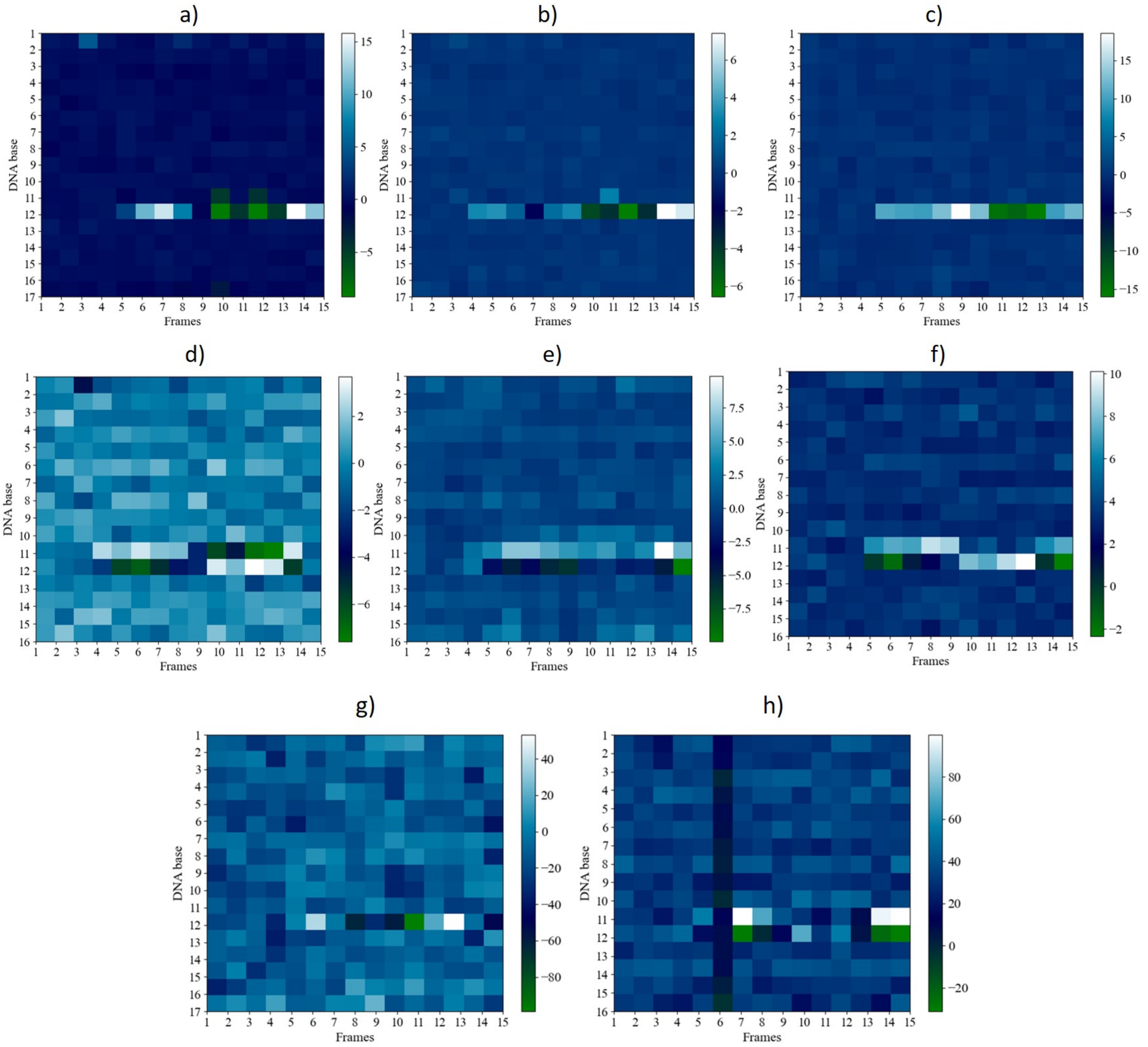


**Figure S4.** Intra-base pair parameters a) shear, b) stretch, c) stagger, g) propel, and inter-base pair parameters d) shift, e) slide, f) rise, and h) twist of uracil-mutated dsDNA for the CPDb scheme were calculated using the CURVES+ program. Here, the dark shades (blue to green) indicate negative values, and the light shades (cyan to white) indicate positive values of uracil-mutated dsDNA.

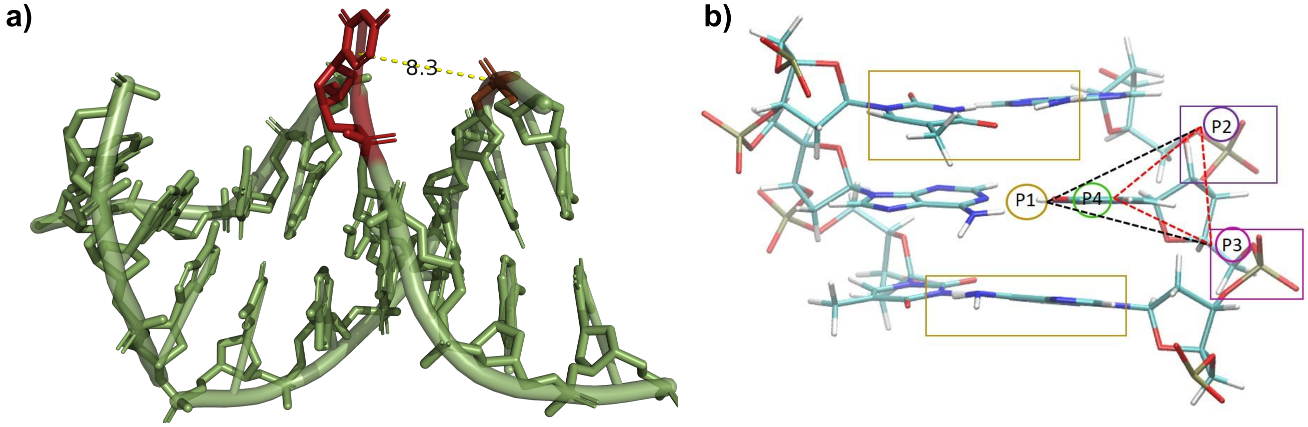


**Figure S5.** Illustration of the DNA feature measurements listed in Table 1; a) Distance measured (Å) in structure T10 (green; two Pymol representations - sticks and cartoon (with transparency)) between the position N1 of the flipped out uracil (red) and the P atom of the closest phosphate group (brown) of the opposite strand's backbone. b) Here, point P1 is the center mass of two flanking base pairs (brown color rectangular box: residues 11, 13, 22, and 24), point P2 (purple rectangular box) and point P3 (magenta rectangular box) are defined by the flanking phosphate group, and point P4 is defined by the target base, i.e. uracil for the flipping process. The dashed lines define the pseudo-dihedral angle formed by four points P1, P2, P3, and P4 measured between the planes passing through P1, P2, P3 (black) and P2, P3, P4 (red).

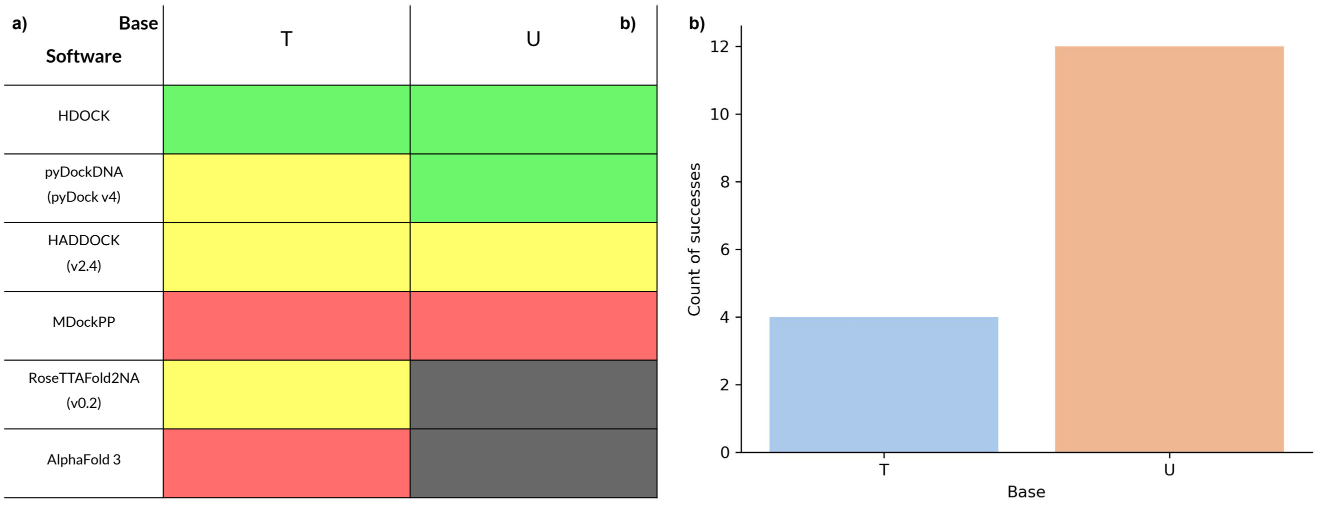


**Figure S6.** a) Table reporting the success of reproducing the PDB:ID 1EMH structure by docking. Inputs given to the software are the separated chains from the complex (for the first four programs): the dsDNA, either with the uracil analog replaced with a thymine or a uracil, and the UDG structure from the crystal structure. Sequences were provided to the last two software packages that do not take structures as input. The color code is the following: green for complete success, yellow for partial success (e.g., the protein-DNA interface is conserved but the substrate residue is not predicted inside the catalytic pocket), red for failure and black for software-inherent inability. See main text for the definition of success. b) pyDockDNA success counts for the DNA substrate containing a uracil compared to when it contains a thymine, justifying the 'partial success' for pyDockDNA with a thymine in a).

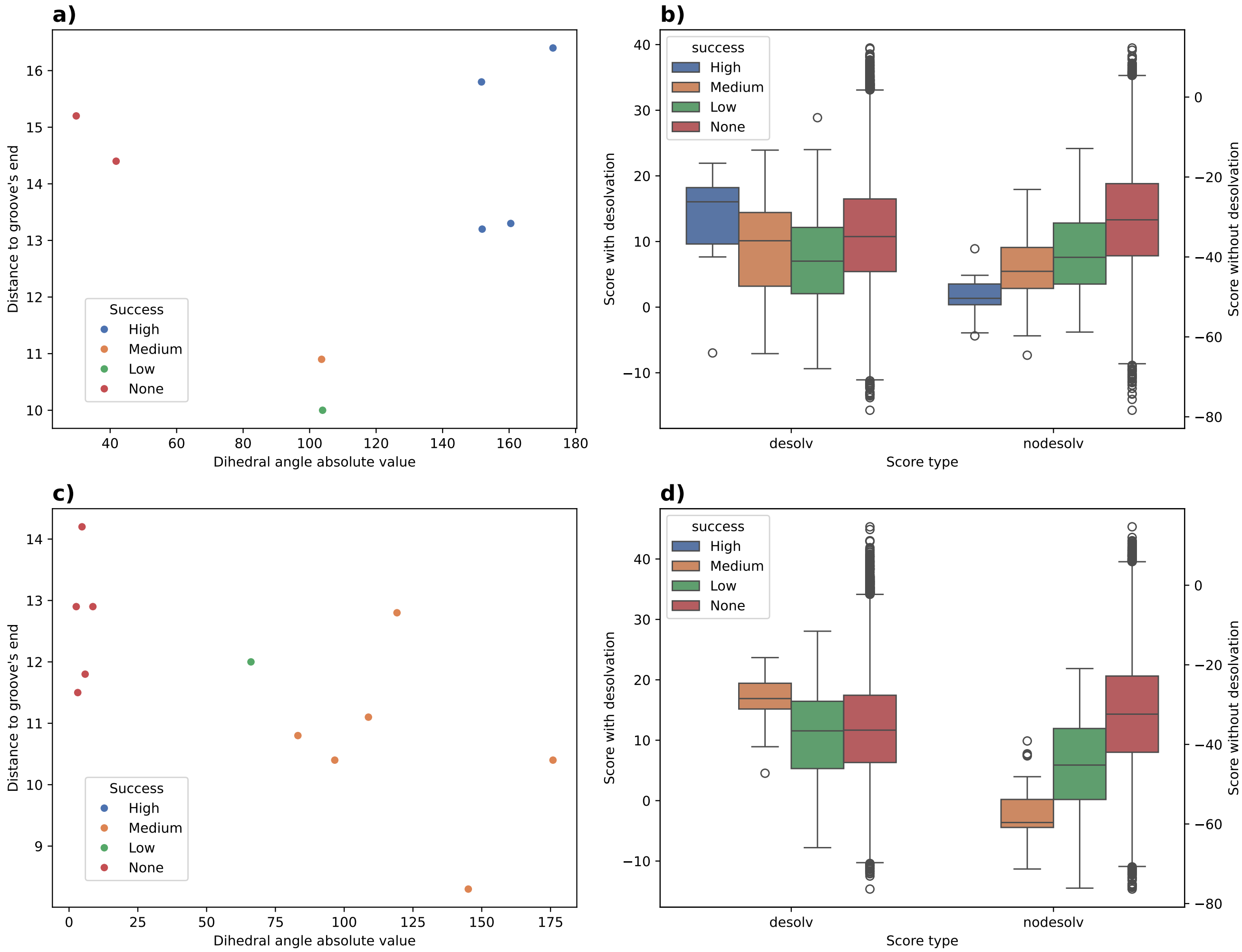


**Figure S7.** Supplementary information on docking quality. Figures a) & b) refer to oligomeric - 'O'- dsDNA, Figures c) & d) are for trinucleosomic - 'T' - dsDNA. For the two dsDNA types, two different types of plots are displayed. The first type, in a) and c), shows scatter plots for docking success as a function of the base flipping angle measured by the dihedral angle and the groove width, represented by the distance from the uracil to the nearest groove's end; the second type, in b) and d), show boxplots of the distribution of pyDockDNA predicted score (with or without desolvation) depending on the finally evaluated docking success.

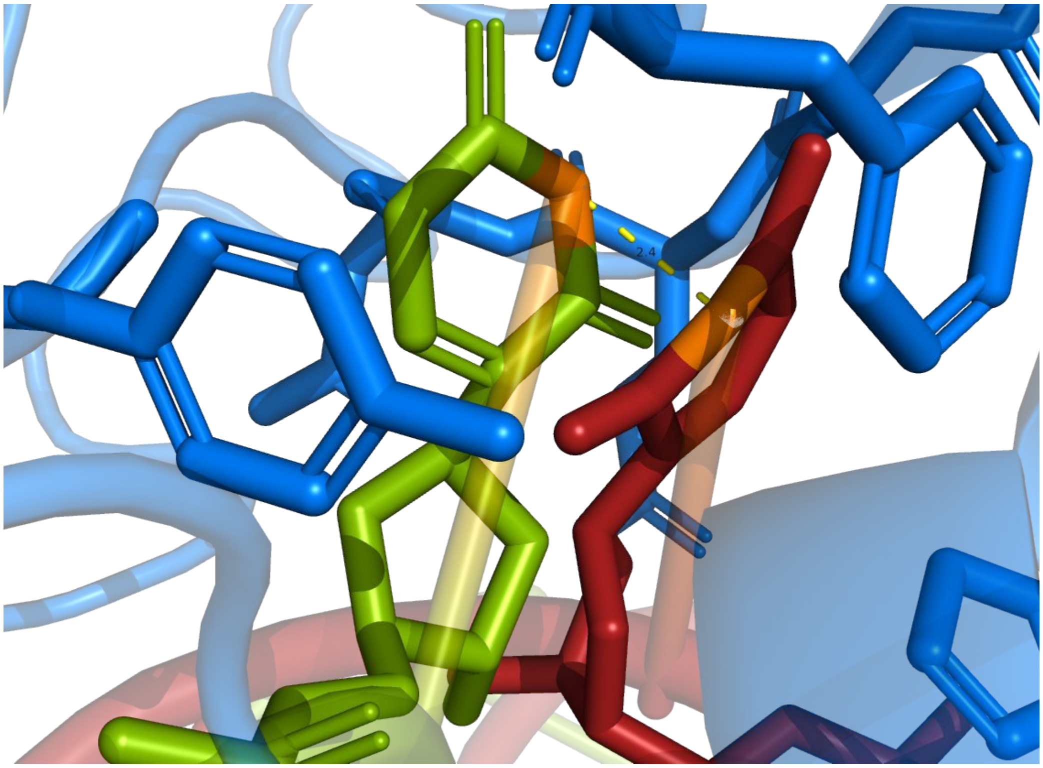


**Figure S8.** Representation of the evaluation for 'O6' best docking conformation by distance measurements. The figure is visualized with PyMOL and respects the following color code and representation: the dsDNA is represented in a cartoon style with their uracil (or uracil analog) as sticks. PDB ID:1EMH dsDNA is in green while the 'O6' dsDNA is in red. UDG is in blue and cartoon except for its catalytic pocket being represented as sticks. Transparency is at 50% applied on the cartoon representation. Atoms located at the endpoint of the measurement lengths are colored in orange. The distance between 'O6' uracil and PDB ID:1EMH uracil analog 2'-deoxypseudouridine is computed between their N3 atoms.

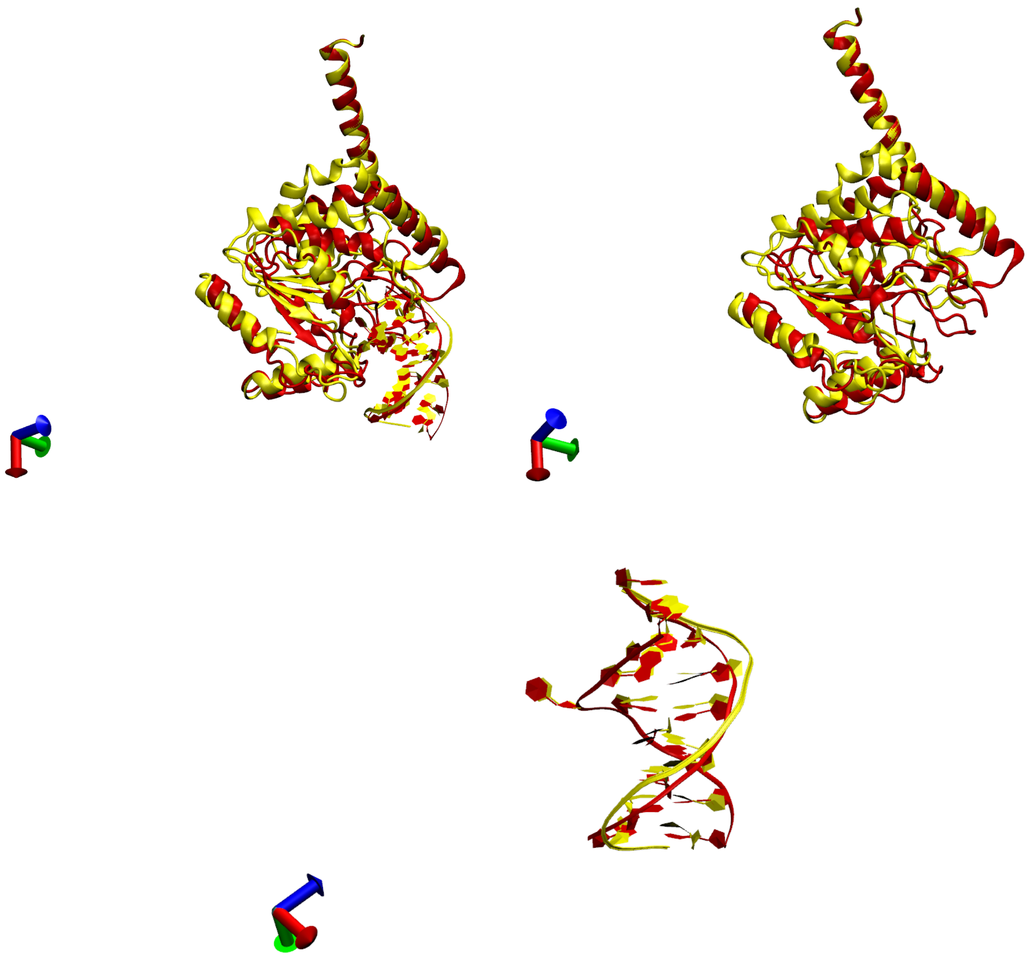

**Figure S9.** Top left: Superimposed structures of the docked UDG-dsDNA complex and the last MD trajectory snapshot (500 ns) of the UDG-dsDNA complex. The red color represents the docked UDG-dsDNA complex, while the yellow color indicates the MD trajectory snapshot of UDG-dsDNA. UDG is shown using the NewCartoon representation, and dsDNA is shown using the NewRibbons representation. Top right and Bottom: Superposition of UDG and dsDNA structures only.

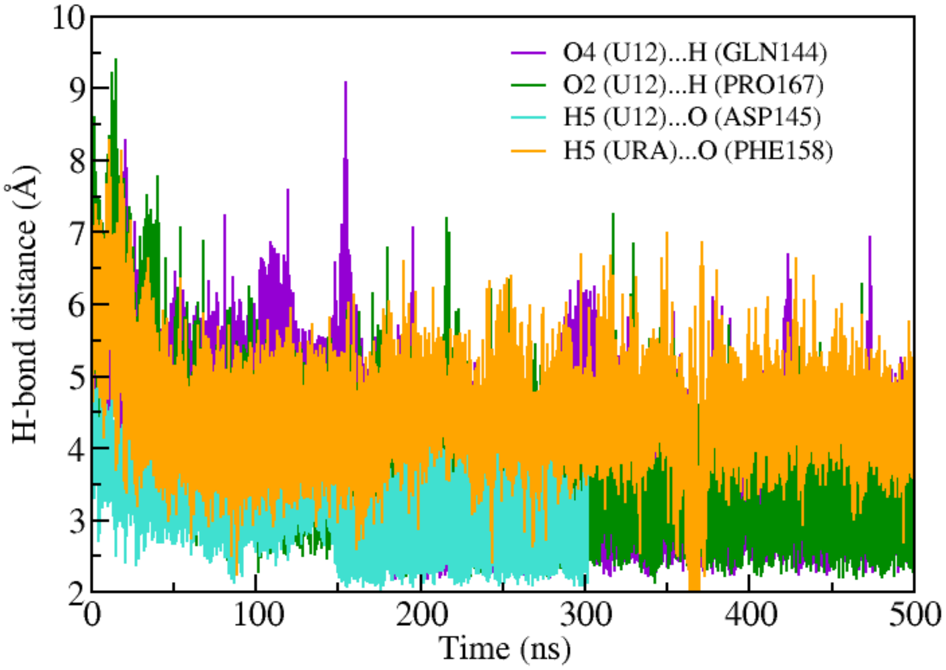


**Figure S10.** Hydrogen bond distance between the uracil and the catalytic sites of UDG enzyme
